# Influence of ARHGAP29 on the Invasion of Mesenchymal-Transformed Breast Cancer Cells

**DOI:** 10.3390/cells9122616

**Published:** 2020-12-05

**Authors:** Katharina Kolb, Johanna Hellinger, Maike Kansy, Florian Wegwitz, Gerd Bauerschmitz, Günter Emons, Carsten Gründker

**Affiliations:** Department of Gynecology and Obstetrics, University Medicine Göttingen, 37075 Göttingen, Germany; katharina.kolb@stud.uni-goettingen.de (K.K.); johannahellinger@gmx.de (J.H.); maike.kansy@stud.uni-goettingen.de (M.K.); florian.wegwitz@med.uni-goettingen.de (F.W.); gerd.bauerschmitz@med.uni-goettingen.de (G.B.); emons@med.uni-goettingen.de (G.E.)

**Keywords:** breast cancer, invasion, EMT, ARHGAP29, RhoA, AKT1

## Abstract

Aggressive and mesenchymal-transformed breast cancer cells show high expression levels of Rho GTPase activating protein 29 (ARHGAP29), a negative regulator of RhoA. ARHGAP29 was the only one of 32 GTPase-activating enzymes whose expression significantly increased after the induction of mesenchymal transformation in breast cancer cells. Therefore, we investigated the influence of ARHGAP29 on the invasiveness of aggressive and mesenchymal-transformed breast cancer cells. After knock-down of ARHGAP29 using siRNA, invasion of HCC1806, MCF-7-EMT, and T-47D-EMT breast cancer cells was significantly reduced. This could be explained by reduced inhibition of RhoA and a consequent increase in stress fiber formation. Proliferation of the breast cancer cell line T-47D-EMT was slightly increased by reduced expression of ARHGAP29, whereas that of HCC1806 and MCF-7-EMT significantly increased. Using interaction analyses we found that AKT1 is a possible interaction partner of ARHGAP29. Therefore, the expression of AKT1 after siRNA knock-down of ARHGAP29 was tested. Reduced ARHGAP29 expression was accompanied by significantly reduced AKT1 expression. However, the ratio of active pAKT1 to total AKT1 remained unchanged or was significantly increased after ARHGAP29 knock-down. Our results show that ARHGAP29 could be an important factor in the invasion of aggressive and mesenchymal-transformed breast cancer cells. Further research is required to fully understand the underlying mechanisms.

## 1. Introduction

With almost 2.1 million new diagnoses (11.6% of incidence) and 627,000 deaths (6.6% of mortality) in 2018, breast cancer is the world’s second most common cancer and the most common cancer experienced by women [1,2]. Due to early detection and improved therapeutic options, 5-year survival rates for local and regional breast cancers are 84–99%. However, only 27% of patients diagnosed with distant metastases survive a period of five years [3].

The first key event in the multi-stage metastasis process is the separation of tumor cells from the primary tumor and spread to the surrounding tissue. Cells acquire the ability to migrate and invade by changing their cytoskeletal organization, cell-cell contacts, and contacts with the extracellular matrix (ECM) and surrounding stroma [4]. Epithelial-mesenchymal transition (EMT) is a dynamic, temporary, and plastic process that goes hand-in-hand with a change from immobile, polarized epithelial cells to mobile, invasive mesenchymal cells [5]. By going through this process, there is a loss of cell–cell adhesion and cell polarity and an increase in mesenchymal properties.

Rho GTPase activating protein 29 (ARHGAP29) was discovered in 1997 as PARG1 (PTPL-associated Rho GTPase activating protein 1) or PTPL1-associated Rho GTPase activating protein with 142 kDa [6]. It has been found to be moderately to highly expressed in many tissues, such as skeletal muscle, the heart, placenta, liver, pancreas, spleen, testes, and parts of the gastrointestinal tract, as well as partly in the kidney, brain, lungs, and skin [6,7,8]. The intracellular protein has, in addition to the domain that gives it its name, a protein tyrosine phosphatase in the C-terminal area for interaction with PTPL, a cysteine-rich domain, and an N-terminal, a ZK669.1a and PARG homology (ZPH) region with homology to the corresponding gene in *Caenorhabditis elegans* [6]. Furthermore, ARHGAP29 has a GTPase-activating protein (GAP) domain, which explains its function as a GTPase-activating protein. ARHGAP29 has a strong affinity for RhoA and weaker affinity for Ras-related C3 botulinum toxin substrate 2 (Rac2) and the cell division control protein 42 (Cdc42) homolog and can specifically regulate the Rho GTPase family member RhoA [6,9]. ARHGAP29 has a suppressive effect on RhoA, and in this way, it has an influence on various processes [6,9,10,11,12]. With an effect on the RhoA/Rho-associated coiled-coil containing protein kinase (ROCK) axis, ARHGAP29 is also found in interaction with Afadin in Rap1-mediated angiogenesis and the migration of endothelial cells [13]. ARHGAP29 has also been described as an effector of Rap2 with an influence on Rho [14]. ARHGAP29 is a suppressor of the RhoA signaling cascade. It is also mentioned in the context of interferon regulatory factor 6 (Irf6)-mediated migration of keratinocytes and endometrial fibrosis by microRNA-1291 [7,15].

It has been shown that ARHGAP29 expression is not only increased in migrating glioma cells and circulating tumor cells but is also associated with an increased tendency to metastasize [12,16]. With regard to an influence of ARHGAP29 on the metastasis cascade, there is initial evidence for the entities of kidney cell and gastric carcinoma [8,12]. A recent publication linked ARHGAP29 expression with transcriptional co-activator Yes-associated protein (YAP) signaling [12]. YAP leads to increased ARHGAP29 expression, and this, in turn, leads to reduced stress fiber formation. If YAP is overexpressed, cytoskeleton reorganization occurs, the dynamics of F-actin/G-actin change, and migration is promoted. YAP is negatively regulated by the Hippo signaling pathway [17]. With regard to breast cancer, YAP has been described in the literature both as an oncogene and as a tumor suppressor [18]. Numerous studies have shown that that increased YAP expression/activity leads to increased expression of mesenchymal markers and also causes morphological changes in the cell that are associated with EMT [17]. Furthermore, several published papers have already shown RhoA-induced regulation of YAP expression [19,20,21].

Little is known about the role of ARHGAP29 in breast cancer. In this study, we analyzed whether changed ARHGAP29 expression influences the invasiveness of mesenchymal-transformed and aggressive breast cancer cells.

## 2. Material and Methods

### 2.1. Cell Culture

The human breast cancer cell lines HCC1806, MCF-7, and T-47D were obtained from the American Type Cell Collection (ATCC; Manassas, VA, USA) and cultured in minimum essential medium (MEM; biowest, Nuaillé, France) supplemented with 10% fetal bovine serum (FBS; biochrom, Berlin, Germany), 1% Penicillin/Streptomycin (P/S; Gibco, Carlsbad, CA, USA), 0.1% Transferrin (Sigma, St. Louis, MO, USA), and 26 IU Insulin (Sanofi, Frankfurt, Germany). The human osteosarcoma cell line MG-63 was purchased from ATCC and cultured with Dulbecco’s modified eagle medium (DMEM; Gibco) supplemented with 10% FBS (biochrom) and 1% Penicillin/Streptomycin (Gibco). To retain the identity of cell lines, purchased cells were expanded and aliquots were frozen in liquid nitrogen. A new frozen stock was used every half year, and mycoplasma testing of cultured cell lines was performed routinely using a polymerase chain reaction (PCR) Mycoplasma Test Kit I/C (PromoCell, Heidelberg, Germany). All cells were cultured in a humidified atmosphere with 5% CO_2_ at 37 °C.

### 2.2. Generation of Mesenchymal-Transformed MCF-7 and T-47D Cells

Mesenchymal-transformed MCF-7 breast cancer cells (MCF-7-EMT) and mesenchymal-transformed T-47D breast cancer cells were generated as described earlier [22]. Briefly, 4 × 10^4^ cells/mL were cultured in a prolonged mammosphere culture (5–6 weeks) in ultralow adherence six-well plates (Corning, Lowell, MA, USA) in DMEM/F12 supplemented with 10% charcoal-stripped fetal calf serum (cs-FCS; PAN-biotech, Aidenbach, Germany), 2% B27 supplement (Invitrogen, Darmstadt, Germany), 1% P/S, 0.5 mg/mL hydrocortisone (Sigma), 5 µg/mL insulin, and 20 ng/mL epidermal growth factor (EGF; Sigma).

### 2.3. Small Interfering RNA Transfection

The breast cancer cell lines MCF-7-EMT (1 × 10^6^ cells/mL), T-47D (1 × 10^6^ cells/mL), and HCC1806 (5 × 10^5^ cells/mL) were seeded in 2 mL of MEM with 10% FBS (−P/S) in a 25 cm^2^ cell culture flask. Cells were transiently transfected with siRNA specific to ARHGAP29 (sc-78491; Santa Cruz Biotechnology, Dallas, TX, USA) in OPTI-MEM I medium (Gibco) with siRNA transfection reagent (sc-29528; Santa Cruz). A non-targeting control was used as a control (sc-37007; Santa Cruz Biotechnology). In order to evaluate the transfection efficiency, a fluorescein-labeled siRNA control (sc-36869; Santa Cruz Biotechnology) was used. After an incubation period of 6 h, MEM supplemented with 20% FBS and 1% P/S was added.

### 2.4. Transwell Co-Culture Invasion Assay

Using the co-culture transwell assay, as described earlier [23], 1 × 10^4^ breast cancer cells were seeded in DMEM w/o phenol red, supplemented with 10% cs-FCS into a cell cultural insert (upper well) with a polycarbonate membrane (8 µm pore diameter, Merck Millipore, Cork, Ireland) coated with 30 µL of a Matrigel^®^ (BD Bioscience, Bedford, MA, USA) solution (1:2 in serum-free DMEM) or gelatin (1 mg/mL in PBS, Sigma). Osteosarcoma cells were seeded (2.5 × 10^4^) in DMEM supplemented with or without 10% cs-FCS into the lower well (24-well-plate). After 24 h, cells were co-cultured for 96 h. Invaded cells on the lower side of the inserts were stained with hematoxylin, and the number of cells in four randomly selected fields of each insert was counted.

### 2.5. AlamarBlue Assay

Transiently transfected breast cancer cells were seeded in 96 wells (1.25 × 10^3^) in DMEM w/o phenol-red supplemented with 10% cs-FBS, and relative AlamarBlue reduction (BioRad, Hercules, CA, USA) was assessed at 120 h. Thereafter, relative AlamarBlue reduction was measured by absorbance readings at 540 and 630 nm using Synergy (BioTek Instruments, Bad Friedrichshall, Germany). Relative AlamarBlue reduction was calculated as indicated by the manufacturer.

### 2.6. Western Blot Analysis

In Western Blot analysis, cells were lysed in cell lytic M buffer (Sigma) supplemented with 0.1% phosphatase-inhibitor (Sigma) and 0.1% protease-inhibitor (Sigma). Isolated proteins (40 µg) were fractioned using 12% sodium dodecyl sulfate (SDS) gel and electro-transferred to a polyvinylidene difluoride membrane (Merck Millipore). Primary antibodies against ARHGAP29 1:2000 (#NBP1-05989, Novus Biologicals, Centennial, CO, USA), AKT1 1:1000 (#9272, Cell Signaling, Danvers, MA, USA), pAKT1 1:1000 (#4058, Cell Signaling), and GAPDH 1:2000 (#5174S, Cell Signaling) were used. The membrane was washed and incubated in horseradish peroxidase-conjugated secondary antibody (GE Healthcare, Buckinghamshire, UK). Antibody-bond protein bands were assayed using a chemiluminescent luminol enhancer solution (Cyanagen, Bologna, Italy).

### 2.7. Real-Time Quantitative PCR Analysis

Total RNA was extracted using an RNeasy mini kit (Qiagen, Hilden, Germany), and 2 µg was reverse transcribed with a high-capacity cDNA reverse transcription kit (Qiagen). Real-time qPCR was performed using an SYBR green PCR master mix kit (Qiagen) and the following primers: ARHGAP29 (forward) 5′-TTG CAG CTC TCC AGG CTA AC-3′, ARHGAP29 (reverse) 5′-AGA TGC TCC TCT TCT GCA CG-3′, and GAPDH (forward) 5′-GAA GGT CGG AGT CAA CGG AT-3′, GAPDH (reverse) 5′-TGG AAT TTG CCA TGG GTG GA-3′. The PCR conditions were as follows: denaturing at 95 °C (2 min), 95 °C (5 s), and 60 °C (15 s) for 40 cycles.

### 2.8. Data-Based Analysis

The publically available TCGA-BRCA dataset from The Cancer Genome Atlas utilized in the survival analyses was downloaded at the TCGA portal [24]. Patient samples of Luminal A molecular subtype (*n* = 480) were selected along the PAM50 annotation.

An analysis of probability of tissue- and process-specific interactions with regard to ARHGAP29 and possible interaction partners was carried out [25]. For this purpose, 987 genome-wide data sets were probabilistically assessed and subjected to a functional integration using the Bayesian method. Data sets for protein interaction were obtained from Bio GRID (Biological General Repository for Interaction Datasets: https://thebiogrid.org), IntAct Molecular Interaction Database (https://www.ebi.ac.uk/intact), MINT (Molecular INTeraction Database: https://mint.bio.uniroma2.it/) and MIPS (http://www.mips.biochem.mpg.de). Data related to miRNA targets were obtained from the MSigDB (Molecular Signatures Database: http://broadinstitute.org/msigdb). The minimum confidence interval chosen was 0.1, and the maximum number of genes considered was 7. The analysis provided a comparison of interaction probabilities between ARHGAP29 and six different interaction partners.

### 2.9. Statistical Analysis

All experiments were performed on at least three biological and technical replicates. Data were analyzed by GraphPad Prism Software version 8.41 (GraphPad Software Inc., La Jolla, CA, USA) using unpaired, two-tailed, parametric t-tests comparing two groups (treatment to the respective control) by assuming both populations had the same standard derivation or with a one-way ANOVA when more than two groups were compared. F-values were recorded, and Dunnett‘s or Tukey‘s multiple comparison test with no matching or pairing between groups was calculated. *p* < 0.05 was considered statistically significant.

## 3. Results

### 3.1. ARHGAP29 Expression in Mesenchymal-Transformed Breast Cancer Cells

**Figure 1:**
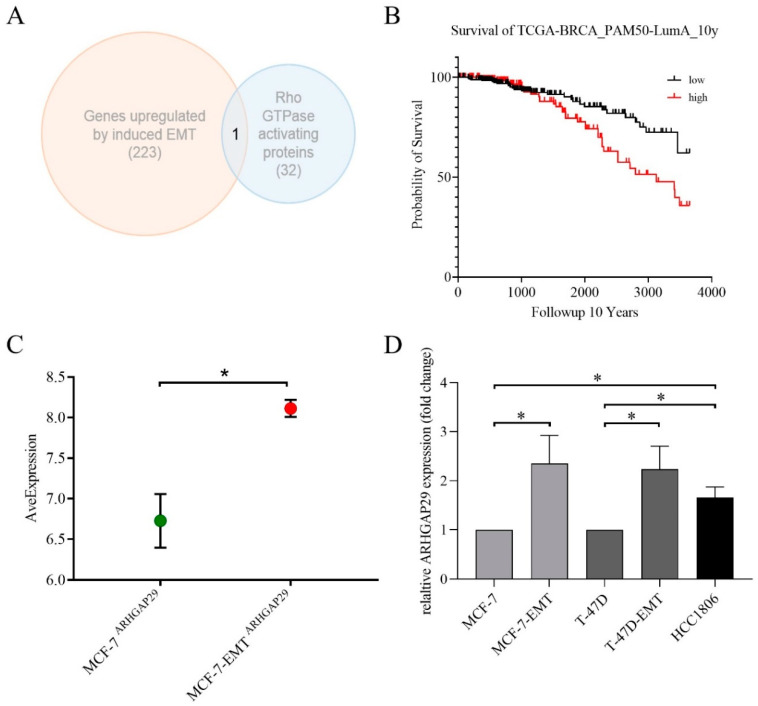
(**A**) Schematic representation of the overlap of microarray data of 223 genes upregulated by induced epithelial–mesenchymal transition (EMT) and 32 considered Rho GTPase-activating proteins. Microarray data from Ziegler et al. [22] were re-analyzed to show the upregulated expression of selected genes. Gene expression data of MCF-7 wild-type cells and mesenchymal-transformed MCF-7-EMT cells were compared. Among the genes examined were 32 genes that had previously been recognized as Rho GTPase-activating proteins. EMT-induced upregulation in MCF-7-EMT cells was found in 223 genes in comparison to MCF-7 wild-type cells. Only the Rho GTPase-activating protein 29 (ARHGAP29) was found to overlap. The relative change in expression (FC, fold change) of genes under consideration was 1.0–4.53. (**B**) Kaplan-Meier survival analysis of the luminal A breast cancer subtype (Threshold = 8 FPKM; ARHGAP29^low^ *n* = 271; ARHGAP29^high^ *n* = 209; Statistical test: Log-rank (Mantel-Cox) test; *p* = 0.0141). (**C**) Evidence of the significantly increased basal expression of ARHGAP29 in MCF-7-EMT cells was found. Basal expression (average expression) of ARHGAP29 in the cell lines MCF-7- and MCF-7-EMT was found. Microarray data from Ziegler et al., 2014 comparing the gene expression of MCF-7 wild-type cells and mesenchymal-transformed MCF-7-EMT cells were examined to investigate ARHGAP29 gene expression. (**D**) Expression of ARHGAP29 in MCF-7, MCF-7-EMT, T-47D, T-47D-EMT, and HCC1806 breast cancer cells. Expression of ARHGAP29 was analyzed by quantitative real-time PCR and normalized to GAPDH expression using at least three biological and technical replicates. Mean ± SEM values are given. Significance was determined with the help of unpaired t-tests; (*) *p* < 0.05.

It has already been shown that mesenchymal-transformed tumor cells are more invasive [22]. The underlying mechanisms by which induction of the epithelial–mesenchymal transition (EMT) causes increased invasiveness is not yet fully understood. A microarray analysis of mesenchymal-transformed MCF-7-EMT breast cancer cells showed that 223 genes were upregulated by the induction of EMT. Among these 223 genes, there was only one GTPase-activating protein (GAP): ARHGAP29 (Figure 1A). Furthermore, expression of ARHGAP29 had an impact on the survival of patients. The 10-year overall survival of luminal A breast cancer patients with high expression of ARHGAP29 was significantly reduced as compared with luminal A breast cancer patients with low expression of ARHGAP29 (Threshold = 8 FPKM; ARHGAP29^low^ *n* = 271; ARHGAP29^high^ *n* = 209; *p* = 0.0141; Figure 1B). We found similar, mostly significant results for the other subtypes of breast cancer (Luminal B: threshold = 4,5 FPKM, ARHGAP29^low^ *n* = 72, ARHGAP29^high^ *n* = 125; *p* = 0.0445; human epidermal growth factor receptor 2 overexpression (HER2+): threshold = 9 FPKM, ARHGAP29^low^ *n* = 59, ARHGAP29^high^ *n* = 15, *p* = 0.0343; Basal: threshold = 8 FPKM, ARHGAP29^low^ *n* = 109, ARHGAP29^high^ *n* = 48, *p* = 0.0505; not shown). However, it is unknown whether ARHGAP29 influences the ability of breast cancer cells to invade and proliferate and which signal cascade is involved.

Mesenchymal-transformed MCF-7-EMT cells showed significantly greater ARHGAP29 expression compared with parental MCF-7 wild-type cells (MCF-7-EMT: 8.114 ± 0.1053 SEM average expression vs. control: MCF-7: 6.728 ± 0.3298 SEM average expression; *p* = 0.0161; *n* = 3; Figure 1B). As confirmed by further gene expression analyses, ARHGAP29 was expressed not only in MCF-7-EMT, but also in mesenchymal-transformed T-47D-EMT cells as well as in the triple-negative breast cancer (TNBC) cell line HCC1806 (MCF-7-EMT: 2, 35 ± 0.5765 SEM relative gene expression (FC, fold change) vs. control; *p* = 0.0473; *n* = 5; T-47D-EMT: 2.235 ± 0.4687 SEM relative gene expression (FC) vs. control; *p* = 0.025; *n* = 6; HCC1806: 1.658 ± 0.2178 SEM relative gene expression (FC) vs. control; *p* = 0.0165; *n* = 5; MDA-MB-231: 7.533 ± 1.145 SEM relative gene expression (FC) vs. control; *p* = 0.0099; *n* = 3; Figure 1C).

**Figure 2:**
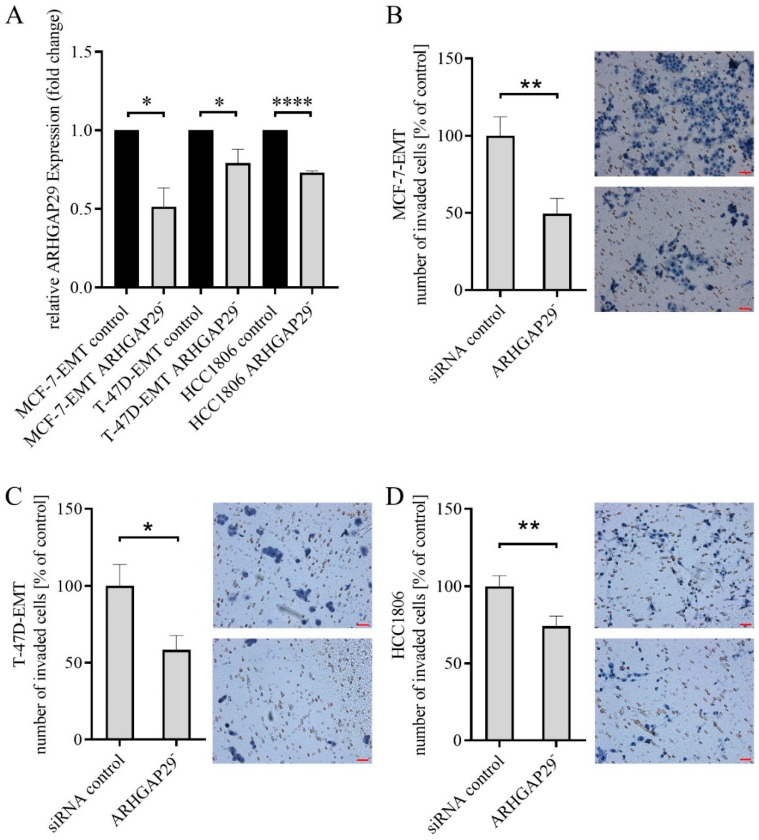
(**A**) Detection of reduced Rho GTPase activating protein 29 (ARHGAP29) expression after transient siRNA transfection of MCF-7-EMT, T-47D-EMT, and HCC1806 breast cancer cells. Breast cancer cells were transiently transfected with ARHGAP29-specific siRNA or non-targeting control siRNA (control). The expression of ARHGAP29 was analyzed by quantitative real-time PCR and normalized to GAPDH expression. The effect of downregulation of ARHGAP29 on the invasion of MCF-7-EMT (**B**), T-47D-EMT, (**C**) and HCC1806 (**D**) breast cancer cells was investigated. Breast cancer cells were transiently transfected with ARHGAP29-specific siRNA or non-targeting control siRNA (control). The invasion of transiently transfected breast cancer cells was analyzed after 96 h of coculture with MG63 osteosarcoma cells. The number of invaded cells in the treatment group was calibrated to the number of invaded cells in the control group using at least three biological and technical replicates. Mean ± SEM values are given. Significance was determined with the help of unpaired *t*-tests; (****) *p* < 0.0001; (**) *p* < 0.01; (*) *p* < 0.05.

We investigated the effects of ARHGAP29 downregulation on the invasion and proliferation of breast cancer cells. For this purpose, ARHGAP29 expression was transiently reduced using ARHGAP29-specific siRNA knock-down (Figure 2A).

### 3.2. Reduced Invasion after Knock-Down of ARHGAP29 Expression

ARHGAP29 is expressed to an increased extent in invasive and mesenchymal-transformed breast cancer cells. After induction of EMT, ARHGAP29 was the only GAP upregulated in both mesenchymal-transformed breast cancer cell lines. Despite this, it remains unclear as to whether ARHGAP29 is involved in the local invasion of tumor cells. Therefore, we investigated whether reduced ARHGAP29 expression influences bone-directed invasion of invasive breast cancer cells. Invasion assays were performed with breast cancer cells transiently transfected with ARHGAP29 siRNA and cells treated with control siRNA.

Compared to cells treated with non-targeting control siRNA, MCF-7-EMT cells with reduced ARHGAP29 expression were significantly less invasive (49.51 cells ± 9.911 cells SEM in % vs. control 99.99 cells ± 12.08 cells SEM in %; *p* = 0.0027; *n* = 18; Figure 2B). T-47D-EMT breast carcinoma cells with reduced ARHGAP29 expression showed significantly reduced invasiveness compared with the control group (58.37 cells ± 9.317 cells SEM in % vs. control 100 cells ± 13.82 cells SEM in %; *p* = 0.0175; *n* = 18; Figure 2C). The invasiveness of HCC1806 TNBC cells with reduced ARHGAP29 expression was significantly reduced compared to the control group (74.09 cells ± 6.54 SEM in % vs. control 100 ± 6.83 SEM in %; *p* = 0.0082; *n* = 30; Figure 2D).

### 3.3. Increased Proliferation after Knock-Down of ARHGAP29 Expression

**Figure 3:**
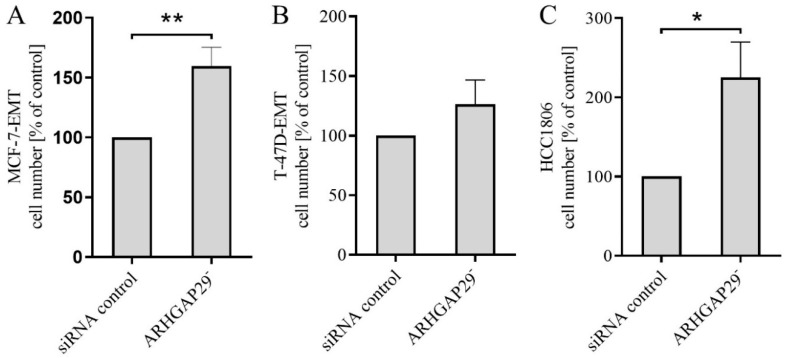
Effect of the downregulation of Rho GTPase activating protein 29 (ARHGAP29) on the proliferation of MCF-7-EMT (**A**), T-47D-EMT (**B**), and HCC1806 (**C**) breast cancer cells. Breast cancer cells were transiently transfected with ARHGAP29-specific siRNA or non-targeting control siRNA (control). After cell culture for 120 h, transiently transfected breast cancer cells were detached and counted using a Neubauer counting chamber. The number of cells in the treatment group was calibrated to the cell number in the control group using at least three biological and technical replicates. Mean ± SEM values are given. Significance was determined with the help of unpaired t-tests; (**) *p* < 0.01; (*) *p* < 0.05.

Since changes in proliferation could influence the results of invasion analyses, the breast cancer cells were analyzed with regard to a possible influence of a reduction of ARHGAP29 on their proliferation. Knock-down of ARHGAP29 expression in MCF-7-EMT breast carcinoma cells led to a significantly increased proliferation rate compared with that of the control (159.5 cells ± 15.78 SEM in % vs. control; *p* = 0.0054; *n* = 5; Figure 3A). In T-47D breast cancer cells, knock-down of ARHGAP29 expression led to a trend towards an increased proliferation rate compared with that of the control (126.3 cells ± 20.35 SEM in % vs. control; *p* = 0.2315; *n* = 5; Figure 3B). The TNBC cell line HCC1806 with reduced ARHGAP29 expression showed a significant increase in the number of cells (225.3 cells ± 44.46 SEM in % vs. control; *p* = 0.0479; *n* = 3; Figure 3C).

### 3.4. Decreased Expression of AKT1 after Knock-Down of ARHGAP29 Expression

**Figure 4:**
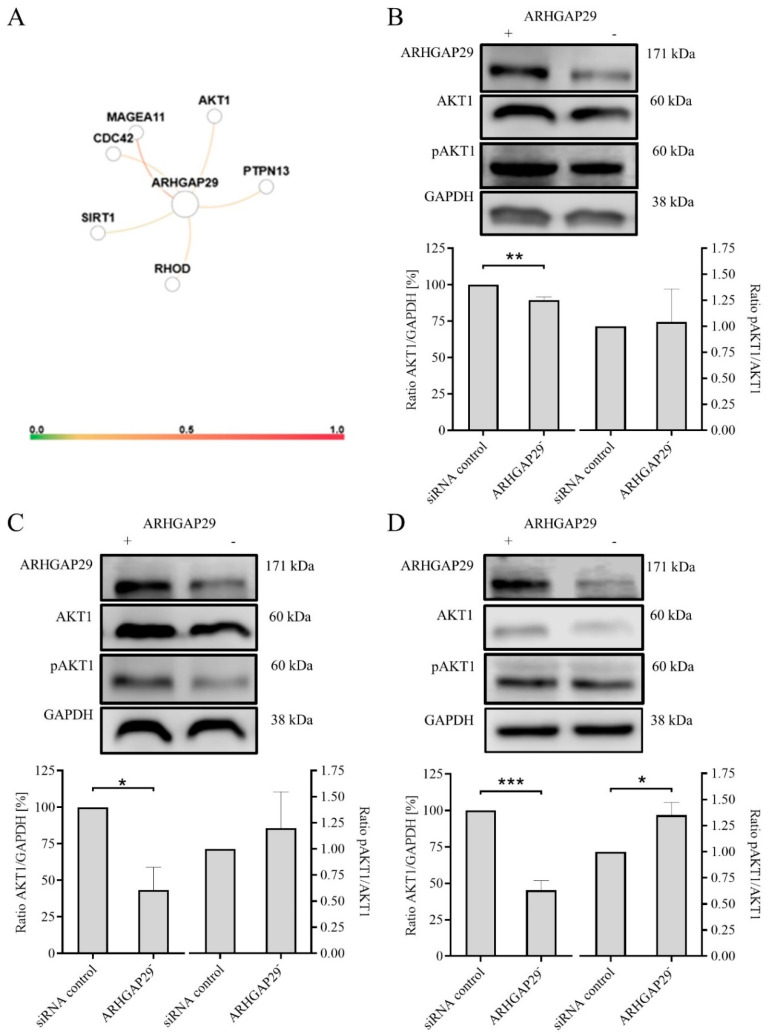
(**A**) Probability of interaction of Rho GTPase activating protein 29 (ARHGAP29) with AKT1, SIRT1, PTPN13, CDC42, MAGEA11, and RHOD. In silico analyses were carried out using the GIANT web server (Genome-wide Integrated Analysis of gene Networks in Tissues, provided by HumanBase, https://hb.flatironinstitute.org/, last accessed on 26 July 2019). A value of 0.1 was chosen as a minimum confidence interval to investigate interactions. The maximum number of genes considered was seven. The color of the connecting lines between interaction partners reflects possible interactions from 0 (no interaction) to 1 (high probability of interaction). The expression of AKT1 and the pAKT1/AKT1 ratio in MCF-7-EMT (**B**), T-47D-EMT (**C**), and HCC1806 (**D**) breast cancer cells after knock-down of ARHGAP29 expression are shown. Breast cancer cells were transiently transfected with ARHGAP29-specific siRNA or non-targeting control siRNA (control). The expression of AKT1 was determined using Western blot analysis and normalized to GAPDH expression using at least three biological and technical replicates. The pAKT1/AKT1 ratio was analyzed as the quotient of pAKT1 versus AKT1 expression. Representative blots for ARHGAP29, AKT1 and pAKT1 are shown. Mean ± SEM values are given. Significance was determined with the help of unpaired t-tests; (***) *p* < 0.001; (**) *p* < 0.01; (*) *p* < 0.05.

In a next step, we looked for interaction partners of ARHGAP29 using in-silico analyses based on GIANT (Genome-wide Integrated Analysis of Gene Networks in Tissues) (Figure 4A). In addition to MAGEA11 (interaction probability 0.4974), RHOD (interaction probability 0.2368), CDC42 and PTPN13 (interaction probability for both 0.2358), and SIRT1 (interaction probability 0.1336), the serine/threonine kinase AKT1 is a possible interaction partner with ARHGAP29 (probability of interaction 0.2358). It is unclear to what extent ARHGAP29 and AKT1 overlap in terms of their effects on Rho signaling or what influence they have on each other. Inspired by these considerations, AKT1 was investigated as a further target in addition to ARHGAP29 for expression analyses to compare breast cancer cells with reduced and normal ARHGAP29 expression and to investigate a possible signal cascade around ARHGAP29. ARHGAP29 and AKT-1 expression was examined in a control group and in breast cancer cells transiently transfected with ARHGAP29-specific siRNA.

In MCF-7-EMT breast cancer cells, a reduction in ARHGAP29 expression was demonstrated 120 h after ARHGAP29 knock-down (63.67 ± 16.23 SEM relative expression ARHGAP29/GAPDH in % vs. control; *p* = 0.0888; *n* = 3; Figure 4B). At this time, AKT1 expression was already significantly reduced compared with that of cells treated with non-targeting control siRNA (89.5 ± 2.062 SEM relative expression of AKT1/GAPDH in % vs. control; *p* = 0.0022; *n* = 4; Figure 4B).

In mesenchymal-transformed T-47D-EMT cells, 120 h after transfection with ARHGAP29 siRNA, there was significantly reduced ARHGAP29 expression (58.5 ± 9.403 SEM relative expression ARHGAP29/GAPDH in % vs. control; *p* = 0.0045; *n* = 4; Figure 4C) as well as significantly reduced AKT1 expression (43.33 ± 15.62 SEM relative expression of AKT1/GAPDH in % vs. control; *p* = 0.0222; *n* = 3; Figure 4C).

The TNBC cell line HCC1806 also showed a significant downregulation of ARHGAP29 expression 120 h after ARHGAP29 knock-down (40.80 ± 10.19 SEM relative expression ARHGAP29/GAPDH in % vs. control; *p* = 0.0004; *n* = 5; Figure 4D) and significantly reduced AKT1 expression (45.25 ± 6.613 SEM relative expression AKT1/GAPDH in % vs. control; *p* = 0.0002; *n* = 4; Figure 4D).

Looking at the activation of AKT, the ratio of pAKT1 to total AKT1 remained the same in MCF-7-EMT cells (1.04 ± 0.32 SEM relative expression pAKT1/AKT1; *p* = 0.9065; *n* = 3; Figure 4B), while it was slightly increased in T-47D-EMT cells (1.20 ± 0.35 SEM relative expression pAKT1/AKT1; *p* = 0.5946; *n* = 3; Figure 4C). In the case of the HCC1806 cells, we observed a significant increase in the pAKT1/AKT1 ratio (1.35 ± 0.12 SEM relative expression pAKT1/AKT1; *p* = 0.0452; *n* = 3; Figure 4D).

## 4. Discussion

Rho GTPase activating protein 29 (ARHGAP29) has a suppressive effect with higher specificity for RhoA compared with Rac and CDC42 [6,9,12]. Via regulation of RhoA, ARHGAP29 not only influences cell–matrix adhesion and the polarity of endothelial cells [9], but it also plays roles in the migration, invasion, and metastasis of various benign and malignant cells [7,8,12,13]. In addition, the expression of connective tissue growth factor (CTGF) is regulated by RhoA activity. Extracellular CTGF drives cell dissemination by regulating cell adhesion, extracellular matrix (ECM) degradation, and regulation of epithelial–mesenchymal transition (EMT) inducing factor transforming growth factor-beta-induced protein (TGFBI; βig-h3) [26]. In this context, the Yes-associated protein (YAP) is known as a transcriptional coactivator of ARHGAP29 [12]. YAP itself has been described as a pro-invasive factor in aggressive and mesenchymal-transformed breast cancer cells [27]. ARHGAP29 expression was increased in various invasive breast cancer cell lines. In addition, ARHGAP29 was the only one of 32 GTPase-activating enzymes with increased expression after epithelial–mesenchymal transformation of breast cancer cells. Furthermore, high expression of ARHGAP29 had a negative impact on the overall survival of patients. Therefore, we analyzed the influence of ARHGAP29 on the invasiveness of breast cancer cell lines. For this, ARHGAP29 expression was transiently reduced using siRNA knock-down. Subsequently, invasion assays in coculture with osteosarcoma cells and proliferation analyses were performed. Furthermore, signaling around ARHGAP29 was examined by analyzing possible interactions with AKT1.

Three breast cancer cell lines with transiently reduced ARHGAP29 expression were examined for their invasive abilities: HCC1806 cells, which are referred to as triple negative due to the lack of expression of progesterone and estrogen receptors and the lack of human epidermal growth factor receptor 2 (HER2) overexpression, and the two mesenchymal transformed cell lines MCF-7-EMT and T-47D-EMT. Increased expression of ARHGAP29 was demonstrated for each of the three cell lines used. Since these cell lines showed increased invasion [22,27,28], it was necessary to check whether reduced expression of ARHGAP29 would lead to reduced invasiveness.

As already described for renal and gastric cancer, ARHGAP29 influences the invasion and metastasis of malignant tumor cells [8,12]. In addition, ARHGAP29 expression is increased in invasive breast cancer cells, and its transcriptional co-activator YAP promotes the invasion of breast cancer cells [27]. We showed that downregulation of ARHGAP29 expression in the breast cancer cell lines examined (HCC1806, MCF-7-EMT, T-47D-EMT) was associated with significantly reduced invasiveness. A reduction in proliferation was excluded as the cause. In contrast, ARHGAP29 knock-down led to increased proliferation with reduced invasiveness, which further reinforced this effect. It could be speculated that ARHGAP29 plays a role in a potential negative feedback loop of YAP signaling.

Previously described effects of ARHGAP29 on the invasion of tumor cells have always been attributed to suppression of the signaling surrounding RhoA [8,12]. In general, however, the role of RhoA in invasion and metastasis in breast cancer is controversial [29]. In addition to an anti-invasive and tumor-suppressive function [29,30,31,32], RhoA also has a pro-invasive role [33,34,35] in breast cancer. New findings show that RhoA can negatively affect the expression of chemokine receptors and thus the metastasis of breast cancer [29]. In general, RhoA controls the formation of stress fibers and is involved in regulatory processes around cell–cell and cell–matrix contacts [36,37,38]. ARHGAP29, in turn, can inhibit RhoA-ROCK signaling and consequently negatively influence stress fiber formation [8,9,11,12,13]. Metastatic tumor cells have a more flexible cytoskeleton than benign cells [39], which means that they can pass through the gaps in the extracellular matrix during metastasis [12,40]. At the same time, MCF-7 breast cancer cells show reduced cell–cell contact and increased migration properties after RhoA reduction, which is comparable to the suppressive effect of ARHGAP29 [32]. In renal cancer cells, the ability to migrate and invade can be significantly increased by overexpression of ARHGAP29 [8]. In connection with reduced invasion after ARHGAP29 knock-down, this indicates a relevant role of ARHGAP29 regarding the invasion of mesenchymal-transformed and invasive breast cancer cells, which can be achieved by favoring a flexible cytoskeleton caused by RhoA suppression but also by less adhesive, more motile cells [8,30,32].

In order to exclude an influence of reduced ARHGAP29 expression on proliferation, and thus a possible causal factor of changed invasion properties, proliferation was analyzed. While no significant effect of ARHGAP29 knock-down on the proliferation of T-47D-EMT cells was detectable, increased proliferation of MCF-7-EMT and HCC1806 cells was found. So far, little is known about how ARHGAP29 or changes in expression or regulation affect the proliferation of cells. Alternatively, increased RhoA signaling could inhibit programmed cell death, thereby increasing the viability of the cells. This protective effect is well characterized [41,42,43]. Mariani et al. [16] discovered that migrating glioma cells not only have increased ARHGAP29 expression but also a down-regulation of proliferation-relevant genes. In several studies, however, the regulation of proliferation by RhoA, which is negatively regulated by ARHGAP29 [9,12,32], has been considered. Zhang et al. (2016) were able to demonstrate a connection between the re-expression of RhoA and increased proliferation of breast cancer cells [44]. In this study, MCF-7 cells were used [44], so that the effect shown is consistent with increased proliferation of MCF-7-EMT cells after ARHGAP29 knock-down and suppression of RhoA. It has already been shown that the regulation of proliferation-relevant genes differs between MCF-7-EMT cells and MCF-7 wild-type cells [22]. However, we can rule out the idea that reduced invasion was caused by changed proliferation in the same direction. Despite an increased proliferation rate, there was a significantly reduced invasion rate.

Our in silico analyses suggested the presence of an interaction between ARHGAP29 and AKT1. AKT1 is a serine threonine kinase [45] and is one of three isoforms of protein kinase B (AKT/PKB), part of the PI3K/AKT signaling pathway (Franke et al. 1995), which is aberrantly regulated in over 70% of breast cancer patients [46]. Although numerous publications have discussed the signal cascade and the role of PI3K and the various AKT isoforms in breast cancer, crosstalk between ARHGAP29 and AKT1 signaling has not yet been reported. We were able to show that reduced ARHGAP29 expression in breast cancer cells is associated with reduced AKT1 levels. Since the activation of AKT1 is dependent on phosphorylation at Ser473 and Th3208 [47], reduced AKT1 expression is not equated with a loss of activity, and a connection with the signal cascade around ARHGAP29 can be assumed. Whereas expression of AKT1 was significantly reduced in all examined cell lines after the ARHGAP29 knock-down, activated pAKT1 in relation to total AKT1 was unchanged or even significantly increased. Since a complex feedback relationship has been found between AKT and Rho activation [48], it does not appear to be unexpected that total AKT levels would decrease after ARHGAP29 knock-down while activation of AKT increases. AKT1 is positively regulated by PAR1 (proteinase activated receptor 1 [49]), TIS21 (tetradecanoyl phorbol acetate-inducible sequence 21 [50]) and caveolin-1 [51] and negatively regulated by PIPP (Inositol-Polyphosphat-5-Phosphatase [52]), miR409-3p [53], PTEN (Phosphatase and tensin homolog [54,55]), SHIP (Signaling inositol polyphosphate phosphatase [56]), PHLPP (pleckstrin homology domain and leucine rich repeat protein phosphatase [57]), and PP2A (protein phosphatase 2A [58]). No interaction with ARHGAP29 is known for any of the factors mentioned. Due to the primarily pro-invasive role of ARHGAP29 and a reduced expression of the two proteins in the same direction, a similar importance of AKT1 to the process of invasion and metastasis of breast cancer cells and possible overlap of the surrounding signal cascades can be assumed. It is astonishing that the majority of the studies on AKT1 have shown an inhibitory effect on the invasion and metastasis of breast cancer [59]. This appears to be in contrast to the significantly reduced invasiveness of the examined cell lines (HCC1806, MCF-7-EMT, T-47D-EMT) in the case of ARHGAP29 knock-down, in addition to reduced AKT1 expression. However, we observed that the proportion of active pAKT1 in relation to total AKT1 remained unchanged or was significantly increased after ARHGAP29 knock-down. In addition to the abundance of publications describing an anti-migratory and anti-invasive role of AKT1, there are also indications that AKT1 has a beneficial effect on the invasion and metastasis of breast cancer [59]. In murine mammary epithelial cells, AKT1 overexpression is correlated with increased invasiveness due to increased MMP (matrix metalloproteinase)-2 expression [60]. Yang et al. (2009) reported that PAR1-AKT signaling promotes the migration, invasion, and metastasis of various breast cancer cell lines [49]. AKT1 knock-down can also reduce the invasiveness of inflammatory breast cancer [61]. Another publication stated that improved invasion properties in malignant cells are mediated by Rap2a-induced upregulation of phosphorylated Akt [62]. ARHGAP29, in turn, has been described as an effector of Rap2 [14]. Whether the results for protein expression in the context of the publications mentioned could represent first indications of a possible signal cascade around Rap2, ARHGAP29, and AKT1 has to be examined in further studies.

Gene deletion of AKT1 resulted in reduced cellular migration in vitro, reduced metastasis of HER2-induced breast cancers, and reduced phosphorylation of the tumor suppressor tuberous sclerosis complex 2 (TSC2) in a murine in vivo model [63]. In addition, AKT1 was shown to have different effects on TSC2 expression and also on RhoA via different types of phosphorylation of TSC2 [63,64]. Liu et al. (2006) stated that constitutive active AKT1 in human breast cancer cells promotes the phosphorylation (Thr-1462) and thus the degradation of TSC2. Therefore, activation of Rho does not occur, which leads to reduced formation of stress fibers and focal adhesions as well as reduced motility, migration, and invasion [64]. This is congruent with the suppressive effect of ARHGAP29 on RhoA [6,9,11,12]. This suggests the possibility for a signal cascade surrounding ARHGAP29, AKT1, and TSC2 with an effect on RhoA, even if the exact mechanism and effect of AKT1 on TSC2 and thus on RhoA remain incompletely understood.

Regarding the contradicting data on the role of AKT1 in the invasion of tumor cells and the poor knowledge of signaling around ARHGAP29 and AKT1, possible crosstalk between ARHGAP29 and AKT1 signaling should be investigated in detail. In summary, we showed, for the first time, that ARHGAP29 influences the invasive abilities of breast cancer cells. Invasion was significantly reduced after ARHGAP29 knock-down. Furthermore, ARHGAP29 plays an inhibitory role in the proliferation of breast cancer cells, whose proliferation can be significantly increased by ARHGAP29 knock-down. Even if the underlying mechanisms remain unclear, it can be ruled out that the effect on tumor cell invasion was caused by proliferation. Our work also shows first indications that ARHGAP29 could interact with AKT1 signaling, which has to be confirmed with further research.
